# Smart glasses use experience of nursing graduate students: qualitative study

**DOI:** 10.1186/s12912-024-01852-w

**Published:** 2024-04-22

**Authors:** Afra Calik, Denizhan Ozkul, Sevgisun Kapucu

**Affiliations:** 1 Nursing Department, Faculty of Health Science, Suleyman Demirel University, Isparta, Turkey; 2 Faculty of Nursing, Gazi University, Ankara, Turkey; 3 Faculty of Nursing, Hacettepe University, Ankara, Turkey

**Keywords:** Smart glasses, Nursing education, Games, Smart technology, Nurse, Serious games

## Abstract

**Background:**

Immersive technologies such as smart glasses can benefit nursing training and clinical practice. In this paper, we explore the views of nursing graduate students about their experience with smart glasses.

**Methods:**

Nursing graduate students (*n* = 13) were recruited using purposeful sampling. First, a virtual reality intervention for hyperglycemia in nursing care was shown. This was an attempt to introduce people to the technology and start discussions about how it might be used in nursing care. After that, participants underwent online interviews. Thematic analysis was used to examine the data.

**Results:**

The study findings indicated that the use of smart glasses as an enjoyable learning experience and immersive games positively affects nursing students. In addition, it was determined that they had negative experiences such as costs, lack of infrastructure, and smart glass side effects.

**Conclusions:**

Smart glasses indicate good usability and availability in nursing education and potential for use in hospital nursing practice.

## Background

Smart glasses in healthcare, especially in nursing education, are essential at the intersection of technology and educational practice. These innovative tools are critical for translating theoretical knowledge into practice by providing nursing students with realistic simulations and interactive learning experiences. This transformation, which started with the first Google Glasses released in 2014, indicates how technology can change daily life. Smart glasses, combined with augmented reality (AR) and virtual reality (VR) technologies, have opened the door to new pedagogical approaches in education [1].

The “Future of Nursing 2020–2030” report highlights nine recommendations critical to transforming nursing education in the modern world. These recommendations include considering access to virtual learning and simulations for students, indicating that educators and teachers should pay special attention to students from diverse backgrounds. Technological developments have enabled the increasing use of wearable technologies, especially in nursing education practices [2–4]. While tools such as smart glasses allow students to improve their independent learning abilities, they also help students master each skill based on their current knowledge [5, 6].

The advantages of using smart glasses include simulations of procedures such as blood transfusion, intradermal injection, and the Leopold Manouver. Students described the simulations carried out thanks to these technologies as realistic and valuable, increasing their learning motivation and quality of education. For example, anaphylaxis scenario simulation using holographic video enriches learning experiences by allowing students to practice in a safe and interactive environment [3, 4, 7, 8].

However, there are also difficulties associated with using smart glasses, such as technological problems and the need to perceive realism in the virtual world. These technologies require regular maintenance and updates and can sometimes result in students encountering simulations that are not as interactive as real-life situations [8, 9]. However, studies conducted in recent years show that overcoming such difficulties is possible with the development of technology [10].

The pedagogical use of smart glasses is of increasing interest because of how they can improve students’ learning processes [11]. Pedagogy requires a framework for structuring experiences that help students create and transfer knowledge. Pedagogical frameworks for how educators and researchers can effectively integrate these technologies to enrich learning and teaching processes are essential for developing models. No single theoretical model is dominant, especially in studies conducted using various digital platforms; instead, approaches exist that use more than one pedagogical theory [12]. There is increasing interest in using virtual reality technologies as pedagogical tools, and research on the contributions of these technologies to the learning process emphasizes the importance of pedagogical approaches. However, there is insufficient evidence on applying pedagogical models to the design and use of virtual learning environments. More research is needed on the feasibility and effectiveness of integrating virtual reality technologies into nursing education [9]. Evidence regarding the feasibility of incorporating this innovative learning and teaching technology into nursing education is lacking. Therefore, before its full-scale integration into the curriculum as a learning, teaching, formative and summative assessment tool, the usability of virtual glass simulation must be investigated, considering the application context. In this context, the study aims to enrich educational practice and improve student learning experiences by providing insights into how smart glasses technologies can be used more effectively in nursing education.

In this study, the students’ views on the smart glasses experience were examined in depth, and the data on the students’ smart glasses experience were collected using the semi structured interview form. In this study, smart glasses technologies enabled students to better perceive the findings by allowing them to experience some nursing practices independently.

## Methods

### Study design

This study employed a thematic analysis method as described by Braun & Clarke [13]. Postgraduate nursing students are clinical nurses and are also pursuing master’s and doctoral education. For this reason, their opinions on the use of smart glasses are important both in terms of education and in the clinic. This research was carried out qualitatively to determine the views of nursing graduate students about their experience with smart glasses. The Consolidated Criteria for Reporting Qualitative Research (COREQ), a suggested reporting system, was used to record the study results [14].

### Study setting

The study was conducted at Hacettepe University of Nursing Faculty, which is situated in Ankara city in the southern region of Turkey. The faculty has 20 students enrolled in internal medicine nursing education. The smart glasses experience was carried out in faculty classrooms with the participation of a researcher. The researcher introduced the smart glasses to each participant, provided them with information about how to use them, and checked for possible side effects. Other researchers interviewed participants online using Zoom after the experience.

### Study participants and sampling

The inclusion and exclusion criteria for this study were as follows:

### Inclusion criteria

- Pursuing postgraduate studies.
- At least 1 year of nurse experience.

### Exclusion criteria

- Newly graduated nurses.
- Not having a postgraduate degree or just starting.

The researcher contacted and utilized a face-to-face technique to tell 20 nursing students about the study and the participant inclusion criteria before selecting study participants for sampling. Only 13 of the potential participants responded to the researcher’s inquiry and met the study’s inclusion requirements.

### Design and development of the game

The treatment of hyperglycemia and nursing care provided by smart glasses are the main topics of this section’s summary of our diabetic patient game. The game’s design is unique and specifically tailored to its intended nursing audience. Two nursing experts helped to develop hyperglycemia treatment. Based on the game’s concept and layout, the colors, text font, and visual identities of the screen elements were created, creating a harmonious composition appropriate for “generation Z”. Wearing smart glasses allowed players to participate in the game’s experiential environment.

- The video game was created using Oculus Quest and Unity 2020.3.3f1 to run on a variety of immersive virtual reality platforms. This game is an extension of earlier projects by Calik et al. The primary goal of earlier incarnations of the project game was to assist with hyperglycemia therapy and nursing care. It talks about helping the nurse care for a patient and orienting the student nurse on their first day at the hospital.
- The Unity Game Engine was used for this project because it makes it simple to create cross-platform video games that can be played on a variety of platforms, including Windows, Mac, Linux, Android, and iOS. Games made in Unity can be smoothly integrated with VR headsets. The accessibility of the game was considered during the design. Head movement is required to play the entire game. The player moves in accordance with the movements of a headset and an object mounted on the Unity XR Rig.

**Fig. 1:**
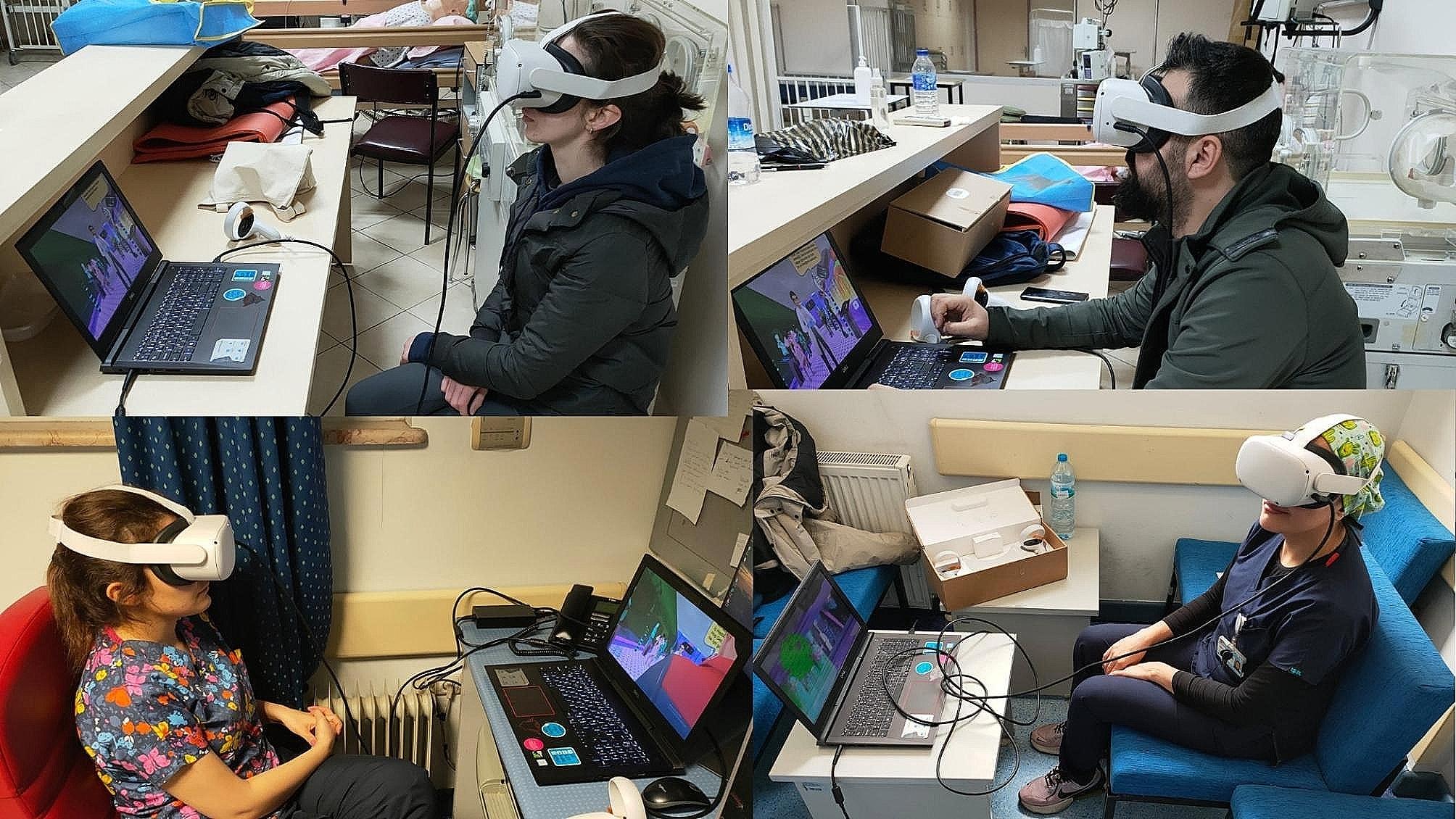
Participants

**Fig. 2:**
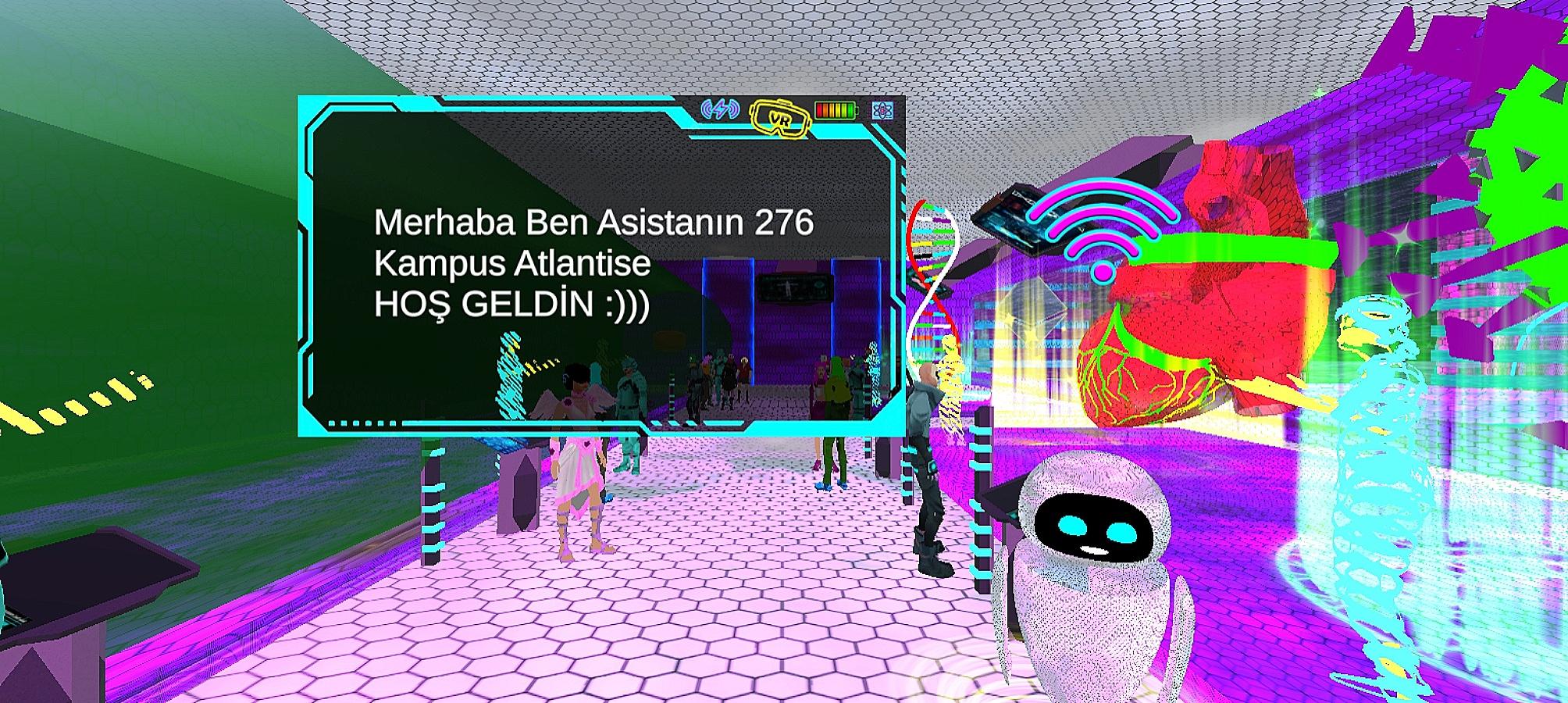
Login Screen

**Fig. 3:**
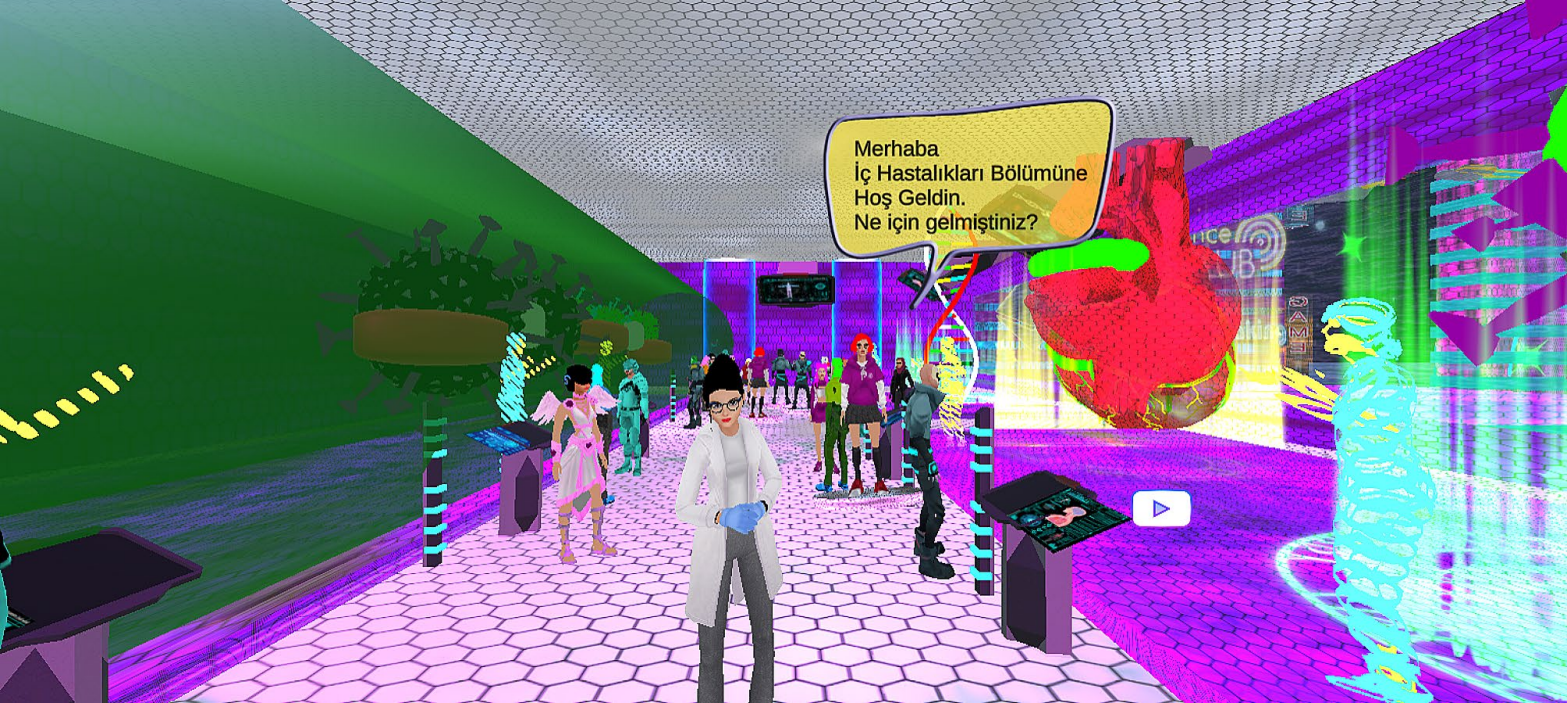
Game information screen

**Fig. 4:**
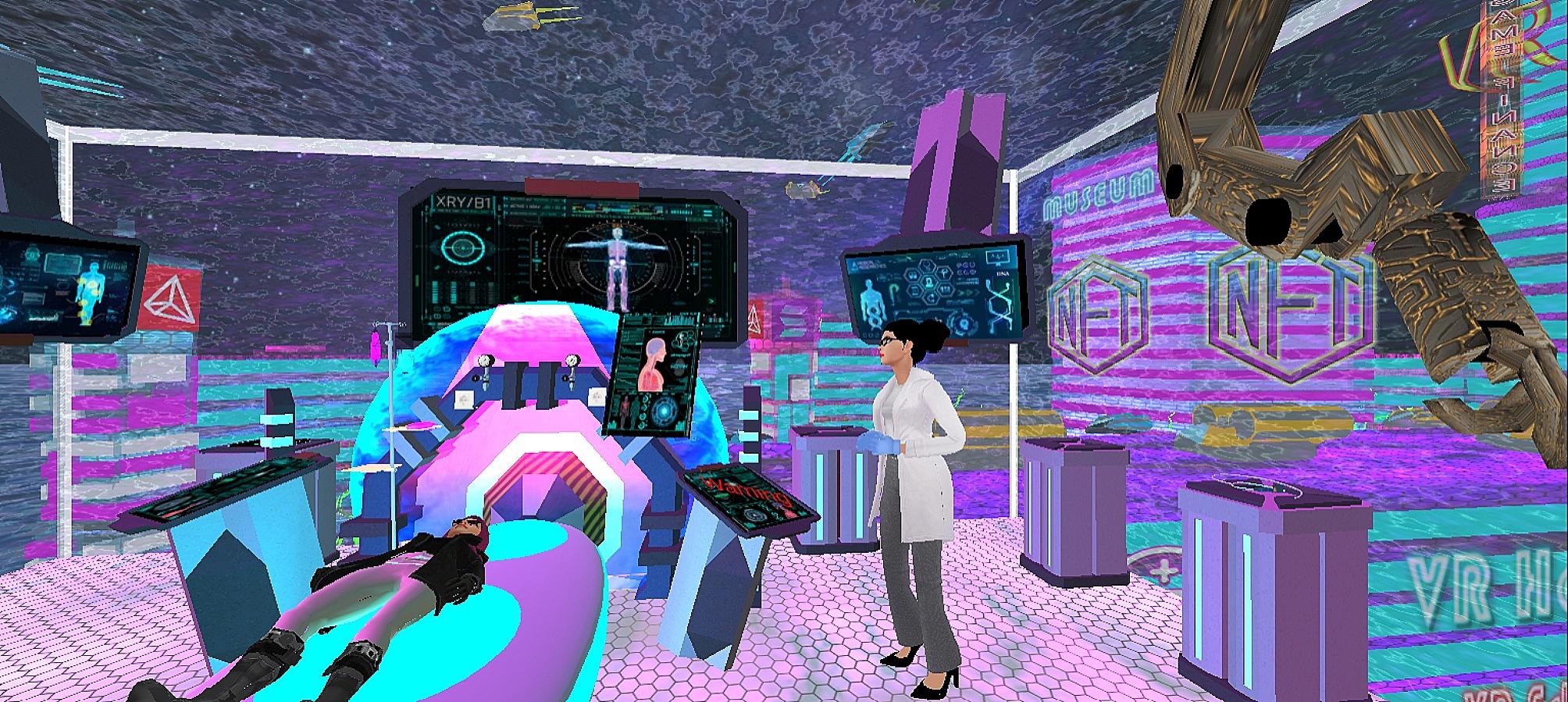
Patient room screen

**Fig. 5:**
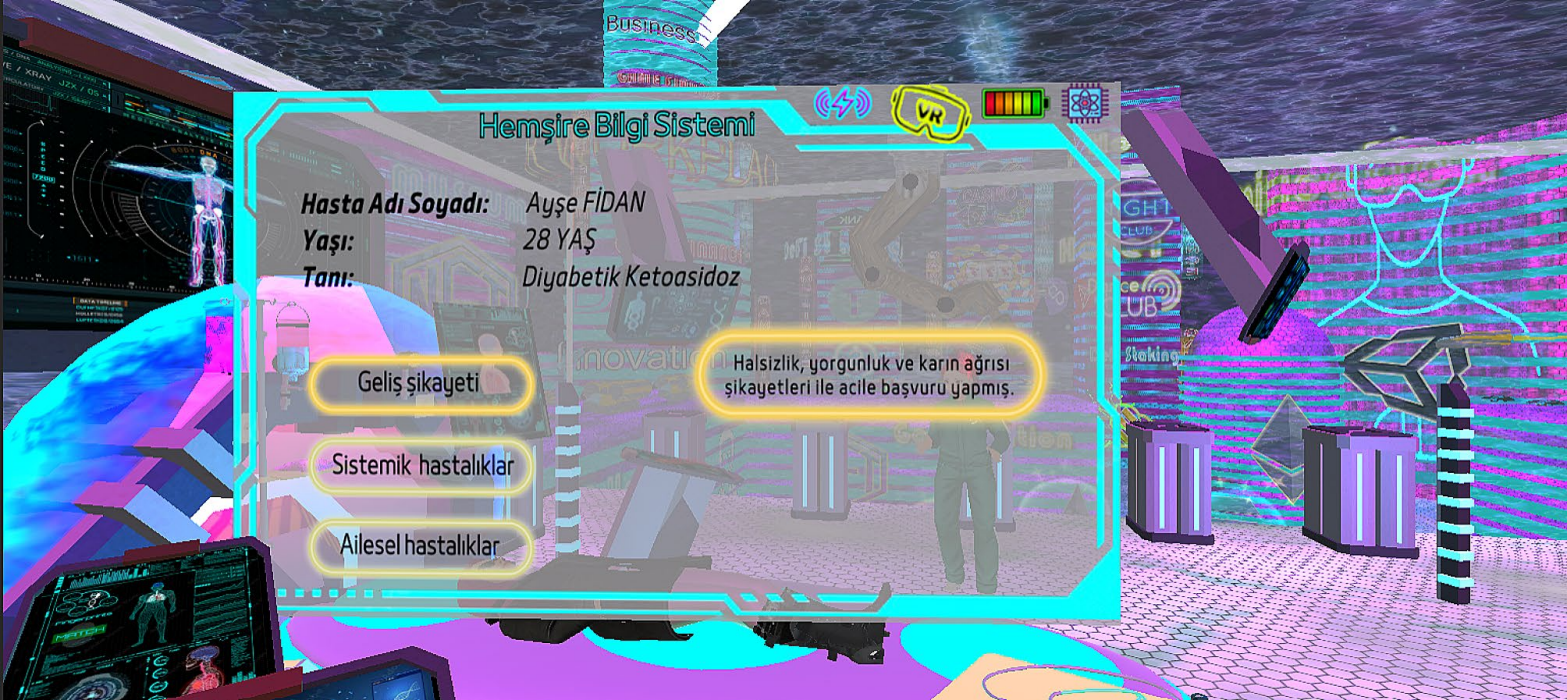
Patient information screen

**Fig. 6:**
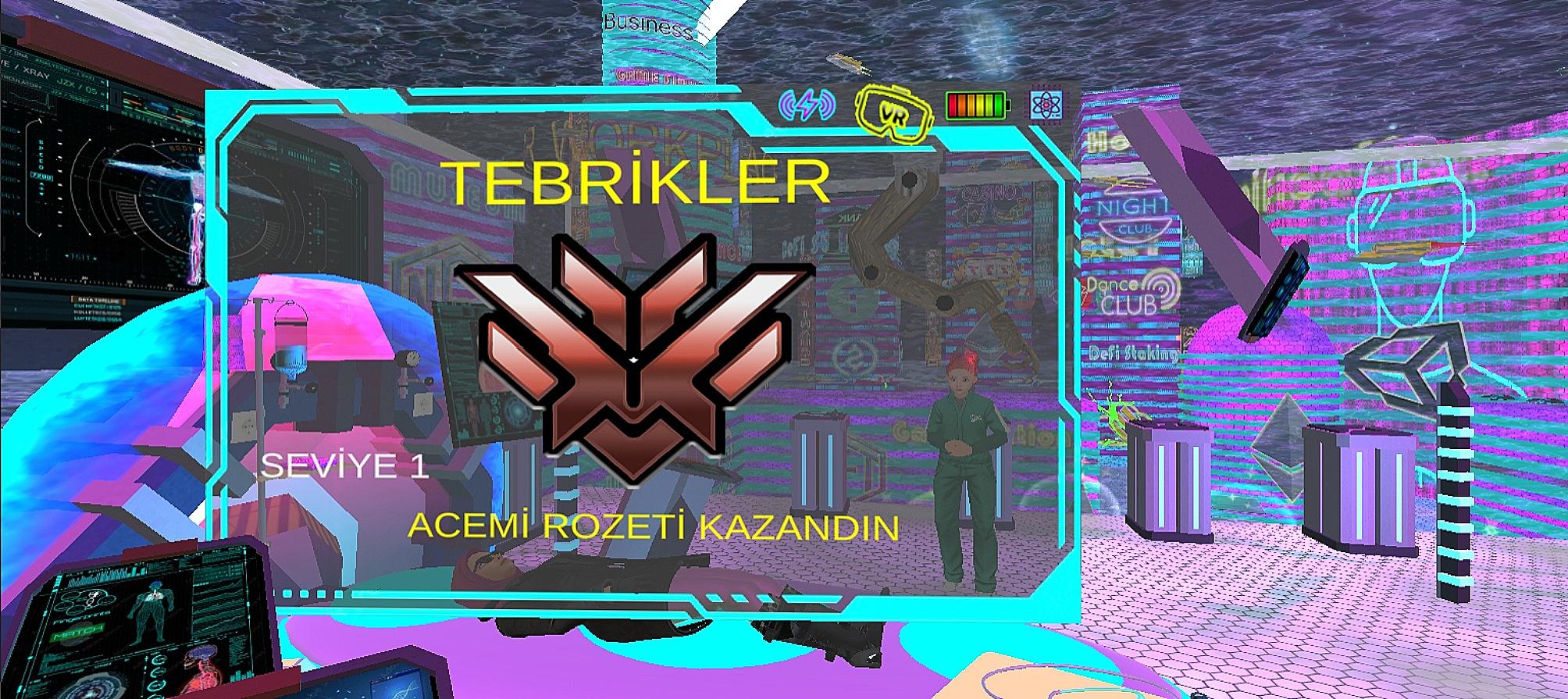
Badget screen

The main technical difficulty with VR in the literature relates to using hand controllers to navigate the virtual environment. Therefore, we researched a video game, and the participants did not use the controllers (Figs. 1, 2, 3, 4, 5 and 6).

### Surveys and questionnaire

When the participants came to the venue, they were first given a sociodemographic characteristics questionnaire consisting of ten questions, which included personal information and previous experiences of the students (Table 1). Afterwards, in-depth interviews were conducted with the participants using the Semi-Structured Interview Form consisting of seven questions. The participants’ opinions about their experiences with the video game using augmented reality technology were determined.

**Table 1:** Interview guides

Regarding the smart glasses experience;	
1.	How did you feel during your experience?
2.	What are the difficulties/conveniences you experience while using smart glasses?
3.	In which areas of nursing (education/clinical) would you like to use the smart glasses?
4.	What are the advantages (s) of smart glasses technologies?
5.	What do you think are the disadvantages(s) of XR technologies?
6.	How would you visually evaluate the environment you experience?
7.	Is there anything you would like to add?

### Data analysis

According to Braun and Clarke’s theme analysis [13], the data were manually evaluated. The process of data analysis was developed step by step. Within 48 h after data collection, interviews were recorded on a digital voice recorder and then verbatim transcribed. Two researchers (A.C. & D.O.) listened to the recorded interviews as part of the initial analysis to obtain a general understanding of the reported use of smart glasses. The data were then read and reread by the two researchers to make sense of the data while looking for patterns and meanings in the context of noteworthy words or phrases. The concepts and codes that overlapped or were similar were compared and debated by the two researchers as a second phase before being combined to create categories. A third researcher analyzed the first classifications and the quotes that accompanied them (S.K.). The aim was to limit the risk of missing relevant units in the condensation process. Finally, the researchers discussed the results, and a consensus was reached on descriptions of the experiences represented by the extracted meaning units.

## Results

The study was conducted with 13 graduate students in the field of internal medicine nursing. There were 10 (77%) females and 3 (23%) males. One participant (7,69%) had a smart glasses experience. Demographics are summarized in Table 2.

**Table 2:** Demographics of students

Demographics	Students		
		*n* = 13	%
Gender	Female	10	77
	Male	3	23
Age		36 ± 10.06	
Degree of Graudate	Master	12	92.3
	PhD	1	7.6
Years of work as a nurse	Mean ± SD	14,3 ± 10.7	
Information about smart glasses technologies	Yes	6	46.1
	No	7	53.8
Previous experience with smart glasses	Yes	1	7.6
	No	12	92.3
Vision problems	Yes	9	69.2
	No	4	30.7
Use of glasses	Yes	8	61.5
	No	6	38.4
Dizziness/vertigo problem	Yes	0	0
	No	13	100
Using technology (0 min-10 max)	Mean ± SD	6 ± 2.81	

### Student experiences

Five themes were identified from the interviews: (i) emotional experiences; (ii) positive experiences of smart glasses; (iii) disadvantages of smart glasses; (iv) future applications; and (v) opinions on game design.

Themes, subthemes, and sample codes are presented in Table 3.

**Table 3:** Sample coding sheet

Themes	Sub-themes	Sample Codes
**Emotional experiences**	**Exciting**	It was an exciting experience.
		The game was enjoyable.
	**Reality Perception**	I felt like I was there.
		It was not lovely to return to the real world after removing the glasses; I loved the environment created.
	**Worry/Anxiety**	I was worried because it was the environment I didn’t know.
		I was afraid of being hit because I was clumsy.
**Positive experiences**	**Immersive Game**	The directions in the game were very accurate.
		It’s really nice to be able to use glasses. It made me wonder why I hadn’t discovered this before.
	**Enjoyable learning experience**	What I saw and read was informative, and it was beautiful.
		Excellent educational material has been prepared to both entertain and attract their attention.
		The directions in the game were very accurate, my theoretical knowledge was transferred here.
	**Glasses compatibility**	I had no trouble adapting while using the glasses.
		I’m nearsighted but I read the articles very easily.
**Disadvantages of smart glasses**	**Fear**	It can create anxiety and fear for people who do not know.
		I am afraid of what can be done because I need a better grasp of new technologies.
	**Cost**	Because glasses are very costly, only some have access to them.
	**Lack of infrastructure**	The standard environment created in the game can be any material, but not in the hospital.
	**Smart glasses side effects**	I experienced headaches and different changes in my field of vision.
		I had an upset stomach.
**Future applications of smart glasses**	**Knowledge and skill acquisition**	It can be used in applications to teach students skills.
	**Personnel and infrastructure support**	It can reduce the number of staff.
		It reduces wastage of time.
	**Nursing care process**	Material use and recognition
		In the care process of immobile patients
		In teaching invasive procedures
	**Hospital Staff Training**	In teaching invasive procedures
		In-service training
		The orientation process of the nurses
**Opinions on game design**	**Character and environment design**	It was nice that it did not look like a hospital, and the characters had an avatar style.
		The absence of a standard hospital environment, the nurse’s clothes were very friendly.
		It would have been better if it was reminiscent of the hospital environment.
	**Visual design**	The design was different and intense.
		Color designs can be changed.
		Vibrant colors forced.

#### Theme 1: emotional experiences

Overall, participants perceived smart glasses as exciting, but some experienced some anxiety. They found the new technology and the experience of wearing glasses for the first time exciting and promising.

### Exciting

Most participants who experienced it for the first time stated that they did not know what to expect, but the environment they saw made them feel happy and excited. They said this: “I was expecting something horror-derived. After that, of course, I accepted it as usual. The game was enjoyable.”

### Reality perception

Participants found the game environment quite realistic. Some participants expressed this situation as follows: “It was not lovely to return to the real world after removing the glasses; I loved the environment created.”

### Worry/anxiety

Few participants stated that they were worried about having an unknown experience, and some were afraid that they would drop objects in the game because they were clumsy in real life.

#### Theme 2: positive experiences of smart glasses

Participants said this was a compelling and entertaining experience overall. They emphasized the interactive and immersive features of the technology and said that smart glasses make learning more memorable than traditional teaching techniques. The value of applying your theoretical knowledge in the game was underlined by the participants.

### Immersive video game

Participants described smart glasses as ‘immersive’ and ‘interesting: “It is nice to use glasses. Why had I not discovered this before?”

### Enjoyable learning experience

Participants explained that smart glasses offer a fun learning environment with the following statements: In such an environment, applying my theoretical knowledge to the patient increased the permanence of knowledge: “Even though I looked around, the constant flow of information in voice and text caused me to stay in the flow of the game.”

### Smart glasses compatibility

In the interviews, participants also talked about the smart glasses’ ease of use. Although none of the participants had prior experience, participants stated that “use it [smart glasses] once, it is so easy to do they will get used to it”:

“I am nearsighted, but I did not have contact lenses. However, I read the articles very easily.”

#### Theme 3: disadvantages of smart glasses

Some participants attributed smart glasses technologies’ disadvantages to being expensive and difficult to access, side effects, and inadequate infrastructure.

### Fear

A few participants said they were alarmed at smart glasses technologies’ innovations. Another stated: It can create anxiety and fear for people who do not know the experience of smart glasses.

### Cost

One of the main reasons why it is rare in Turkey is that the smart glasses technologies it offers are costly. Some of the participants stated that it is challenging to use high-cost glasses in a hospital environment.

### Smart glasses side effects

A few of the participants said that they had complaints of dizziness and nausea while using smart glasses.

#### Theme 4: future applications of smart glasses

To provide an idea for future studies, some participants presented their views.

All participants recommended using smart glasses in the future, mainly in nursing care, compliance with the hospital, and to encourage nurses to support the treatment and procedures applied to the patient. Smart glasses were also recommended for developing nurses’ knowledge and skills.

### Knowledge and skill acquisition

Participants emphasized that smart glasses would contribute to developing skills, especially in students.

### Personnel and infrastructure support

A few participants stated that using smart glasses will save time orienting patients and newly arrived nurses, thus providing staff support.

### Nursing care process

The use of smart glasses in health education was a key discussion point in all the interviews. For example, increase the skills of nurses, especially in invasive procedures that require skill, and facilitate the nursing care process of immobile patients.

### Hospital staff training

The participants state that giving information by using smart glasses in in-service training and increasing skills in invasive procedures with smart glasses without harming the patient will strengthen the nurse. These expressions: It can be taught very well without harming the patient for those training for the first time or who will adapt to the hospital environment.

#### Theme 5: opinions on game design

The participants’ opinions about the game design concerned the hospital environment, character design, and visual objects.

### Character and environment design

While some participants described the hospital environment as a free environment without standards, others criticized the game environment as being very active and not reflecting the hospital environment.

### Visual design

Many participants found the color scale used in the game lively and suggested making it soft.

## Discussion

This study explored graduate nursing students’ views of using smart glasses in nursing healthcare following a brief intervention to promote nursing care in hyperglycemia treatment. Participants generally strongly emphasized the novelty, ease of use, and enjoyable learning experience of smart glasses.

In our study, we selected nursing students enrolled in a graduate program. Our aim in selecting the sample was that we thought that nurses with clinical experience would add a different perspective to the use of virtual glasses. We also thought that they would bring more suggestions and comments regarding the aspects that could be useful and improved in hospital education and postgraduate education, and indeed, they did. We had participants who felt better because the environment they experienced was different from the hospital environment or because they did not find it realistic in terms of design. According to the literature, the opinions of students who have more clinical experience than undergraduate students are more meaningful [8], and doing so in small groups, such as graduate students instead of crowded undergraduate students, is more effective with virtual glasses. associated it with having experience [15].

Interactive, immersive environments such as AR, VR and XR have generally created positive emotions in users. Participants generally emphasized the novelty, ease of use, and enjoyable learning experience of XRs. In one study, users experienced more positive emotions than in computer-based simulations [16]. Another study stated that nursing students showed great interest in their smart glasses experience and were encouraged to reduce their anxiety [17]. Although the immersive nature of smart glasses enhances experiences in many contexts, participants said that they feel worried and anxious. We attribute this to the fact that smart glasses technology is new, and the participants need to gain more knowledge of this technology.

Smart glass technology is a rich, interactive, and engaging educational strategy supporting experiential learning [18]. In our study, participants emphasized their experience as an immersive, fun learning experience. In previous studies, students stated that using smart glasses technology to view 3D structures seems more motivating and exciting than traditional methods such as books [19, 20]. An opposing view stated that the immersive experience of being in a simulated reality overshadowed the acquisition of cognitive skills. For example, Moesgaard et al. (2015) described this as the place where study participants reported that they were “too fascinated by the [virtual] environment to notice the information presented to them.” [21]. Even though one participant in our study found the environment he was experiencing gripping, the statement, “When I turned my head to a different place, the information continued to come in visual and written information, and this helped me to break away from the flow,” showed that it was immersive and focused on the game. In addition, the participants stated that adapting to smart glasses technology is comfortable. In this case, it can be associated with the fact that we did not include any interaction area in the game. Because smart glasses technologies are costly in Turkey, this study does not receive financial support. However, given the rapid development in the field, we expect the smart glass hardware landscape in our country to change quickly.

Motion sickness and nausea are drawbacks of smart glasses [22]. We asked whether motion sickness and nausea are caused by novices’ attempts to become accustomed to the technology or whether they linger over time and can potentially impair learning or education. A few participants in our survey claimed to wear glasses and suffer from environmental sickness. consequences may vary depending on how real-world and virtual surroundings are combined in AR devices, but this should lessen negative health consequences, including dizziness and blurred vision, in VR applications [23].

The usage of the content created for trainers is challenging due to the high cost of smart glasses technology [22]. The participants acknowledged the advantages of these technologies but claimed that because of their high cost, accessing them was challenging. Its usage for independent study at home is hindered by high costs, such as the price of the virtual dissection table. Due to economies of scale, some technologies may be able to lower costs [24]. Although these technologies have received excellent review, it is still unclear who will be responsible for paying these expenses. Therefore, when utilizing these technologies, cost-effectiveness should be considered.

The use of smart glasses in nursing care was suggested by the participants to enhance patient care and treatment. Since the idea of utilizing immersive technology in nursing education has still been relatively undeveloped, it was simpler for researchers to develop new concepts, introduce improvements, and make contributions to the profession. Many academics are interested in the concepts, features, technical principles, tools, application scenarios in various sectors, existing issues, and prospects of smart glasses technology. With the integration, in-depth interaction, and coexistence of various temporal and spatial objects, smart glasses are now being incorporated into the educational sector [25]. In addition to skill areas such as classroom nursing education, clinical nursing, intensive care nursing, and intravenous procedures, this approach has obvious technical advantages in the orientation process of new nurses and has unlimited potential development and application areas.

While respondents generally described smart glasses as a fun visual design, they found the technology suitable for younger demographic information and too mobile for middle-aged adults. Technology use, exposure, and unfamiliarity can affect opinions about design [25]. In addition, the personal differences of individuals in the learning environment can distinguish design evaluations in the experience environment, which is quite normal.

### Limitations

The reason for this was that the game developed for smart glasses was about diabetes. Therefore, a purposive sample was used, and students enrolled in the internal medicine nursing graduate programme were included in the study.

## Conclusion

In the education of nursing graduates, there are great opportunities for the use of smart glasses. Smart glasses games can play a supportive role in the transfer of graduate knowledge and skills to the clinical environment and education.

These results showed that the nurses working in the clinic had no difficulties using smart glasses and even enjoyed the experience, even though they were not very familiar with the technology used in education. They interpreted the smart glasses as an opportunity to practice nursing processes and skills in a variety of settings. For example, for nurses starting out in the clinic, working in intensive care, and in nursing education. There is a need to integrate smart glasses into the training of nurse educators and hospital administrators.

## Data Availability

Not applicable.

## Declarations

### Interview guide

Interview used was developed for this study and has not previously been published elsewhere.

## Abbreviations

GPS: Global Positioning System
